# Retinal Organoids: A Next-Generation Platform for High-Throughput Drug Discovery

**DOI:** 10.1007/s12015-023-10661-8

**Published:** 2023-12-11

**Authors:** Hongkun Zhao, Fei Yan

**Affiliations:** 1 Key Laboratory of Yunnan Province, Yunnan Eye Institute, Affiliated Hospital of Yunnan University, Yunnan University, Kunming, Yunnan China; 2 Department of Pathology and Pathophysiology, Faculty of Basic Medicine School, Kunming Medical University, 1168 Yuhua Street, Chunrong West Road, Chenggong District, Kunming, Yunnan, 650500 China

**Keywords:** Retinal Organoid, Drug Discovery, Bioengineering, Stem Cells, Organ-on-a-chip

## Abstract

Retinal diseases are leading causes of blindness globally. Developing new drugs is of great significance for preventing vision loss. Current drug discovery relies mainly on two-dimensional in vitro models and animal models, but translation to human efficacy and safety is biased. In recent years, the emergence of retinal organoid technology platforms, utilizing three-dimensional microenvironments to better mimic retinal structure and function, has provided new platforms for exploring pathogenic mechanisms and drug screening. This review summarizes the latest advances in retinal organoid technology, emphasizing its application advantages in high-throughput drug screening, efficacy and toxicity evaluation, and translational medicine research. The review also prospects the combination of emerging technologies such as organ-on-a-chip, 3D bioprinting, single cell sequencing, gene editing with retinal organoid technology, which is expected to further optimize retinal organoid models and advance the diagnosis and treatment of retinal diseases.

## Introduction

The retina is a layer of sensitive neural tissue lining the inner wall of the eyeball, playing an extremely important role in human visual perception. The retina contains numerous photosensitive cells, such as rod and cone cells, which can sense light stimuli and convert them into neural impulses that are transmitted to the visual cortex in the brain [1].

Retinal diseases pose a major public health threat worldwide. As part of the central nervous system, the retina lacks regenerative capacity and any damage often leads to permanent, irreversible vision loss. Globally, retinal diseases are a leading cause of blindness, affecting millions of patients. Statistically speaking, major retinal diseases include age-related macular degeneration impacting approximately 8.4 million people worldwide, glaucoma afflicting 4 million, diabetic retinopathy in 2.6 million [2], and retinitis pigmentosa diagnosed in over 1 million [3]. With the aging of the global population, the number of patients affected by retinal diseases is on the rise. These vision impairments severely compromise quality of life at the individual level. Additionally, vision loss from retinal diseases has tremendous economic impacts on society in terms of healthcare costs, loss of productivity, and reduced independence. In summary, retinal diseases represent a major challenge for aging populations that necessitate novel therapies to preserve vision and reduce global blindness [2]. Advancing new treatment options is critical to reduce the individual, societal and economic burdens of these blinding diseases. According to a study on the economic burden of vision loss in 7 provinces or cities in China, the direct medical costs of vision impairment were about 15.9 billion RMB, and the indirect socioeconomic losses were as high as 81.3 billion RMB.

Despite the urgent and growing public health need, treatment and management of retinal diseases remains challenging. Although research institutions are actively pursuing regenerative strategies, most potential therapies have yet to reach clinical translation. Currently, patients predominantly rely on traditional approaches focused on prevention or slowing disease progression, which often demonstrate poor efficacy. There is a definitive, unmet need for novel therapeutics that contrasts sharply with the relatively slow progress in retinal drug research and development. Statistics reveal the inefficiency of current drug development pipelines. As of 2015, only 13.8% of drug candidates across all categories successfully progressed from Phase I trials to marketing approval. For those few drugs eventually approved, average research and development costs reached $5 billion. These data highlight the remarkably low efficiency of conventional pharmaceutical research and development processes. In summary, while retinal diseases are rapidly increasing worldwide, new treatment options are slow to emerge and face immense clinical translation hurdles. There is an urgent need to enhance the efficiency of retinal drug development to deliver innovative therapies to patients. Improving success rates for clinical translation would significantly accelerate development of novel treatments to preserve vision and combat global blindness.

In preclinical retinal disease research, conventional models utilize two-dimensional (2D) cell cultures and animal models. However, these approaches face considerable limitations for screening and developing new therapeutic drugs. 2D cell cultures fail to reconstitute the complex in vivo physiological environment, lacking diverse cell types, tissue architecture, and mechanical and biochemical signaling dynamics. Animal models, due to interspecies differences, often do not accurately reproduce human disease pathogenesis, with risks of false negative or false positive outcomes. Drug efficacy and toxicity may substantially differ across species. The key innovations for improved retinal disease models are better biomimicry of the human retinal microenvironment by incorporating multiple cell types in three-dimensional architectures and considering mechanical and biochemical factors [4–7]. Meanwhile, emerging technologies should be leveraged to increase assay sensitivity for elucidating disease mechanisms and assessing drug responses. Ideally, next-generation retinal disease models will integrate human cell sources, 3D tissue structure, and microenvironmental cues to improve clinical relevance [8]. Incorporating microfluidics, biosensors, and imaging modalities can enhance resolution for unraveling pathophysiological processes and evaluating drug effects [9–13]. Improved biomimicry and sensitivity in preclinical retinal models will accelerate translation of new therapies to patients with vision-threatening diseases.

Human stem cell-derived retinal organoids (ROs) offer tremendous potential as next-generation models that closely mimic in vivo retinal structure and function [8]. These 3D tissue constructs contain diverse cell types and can recapitulate developmental processes in vitro. With microfluidic technologies, organoids can model biochemical and mechanical features of the retinal microenvironment [9–13]. These biomimetic attributes enable high-throughput, efficient drug screening for efficacy and toxicity. RO allow evaluation of individual donor variations and improved prediction of human responses, bypassing limitations of animal models for more direct translation to clinical trials [14]. Overall, RO significantly enhance the success rate and efficiency of drug development by shortening research and development timelines and lowering costs. These 3D, human models represent a promising new platform for pharmaceutical screening and translational medicine. By integrating stem cell and tissue engineering technologies, single cell sequencing, gene editing, viral transduction etc., RO have achieved unparalleled biomimicry of human retinal structure and function [13, 15–20]. Their integration of multiple cell types, 3D architecture, microenvironmental cues, and potential for high-throughput assays accelerates preclinical drug discovery. RO thus provide an advanced solution to current inefficiencies in retinal disease research and therapy development.

## Retinal Organoid Protocols

RO first appeared in 2011 when Meyer et al. researchers first induced optic vesicle-like structures from human embryonic stem cells, expressing early retinal markers and differentiating into photoreceptor-like cells [21. The Sasai lab found that optic cup development is a self-guided process, independent of external structural influences. Their models contained the 6 major neural types and 1 glial type of the retina, recapitulating many aspects of retinal function including morphogenesis, interkinetic nuclear migration, and apicobasal polarity [22–23]. This opened the door to RO induction techniques and gradually unfolded a great deal of research on retinal related diseases in 3D models.

In this model, the percentage of cone cells was low. Although it could not perfectly reproduce the mouse retina, further optimization first achieved the generation of ROs derived from human (h)ESCs [24]. This method is completely based on 3D culture, starting from a single cell suspension, human embryonic stem cells are seeded in equal numbers into V-shaped wells of 96-well plates, rapidly aggregating to form embryoid bodies (EBs). These EBs undergo neural induction to form optic vesicles, followed by continued suspension culture to differentiate into laminated ROs. Therefore, human stem cell-derived RO models have longer culture times, mainly due to natural interspecies differences in gestation periods between humans and mice. hiPSCs are grown close to confluence and made into small floating aggregates via chemical or mechanical means in suspension, forming EBs, undergoing neural induction, then ROs are [25–26] selected after which long term suspension culture allows formation of laminated and mature ROs. Researchers then developed an alternative approach, allowing hiPSCs to grow to confluence rather than generating small floating aggregates, maintaining adherent culture. Removing FGF2 from the culture medium initiated spontaneous differentiation, followed by promotion of neural induction and neuroepithelial formation [27–28]. After inducing formation of mature retinal tissues, researchers began testing retinal responses to light. Zhong et al. method first presented fully laminated 3D iPSC-derived retinal tissues, which also produced more developed light-responsive outer segment structures [25].

**Fig. 1:**
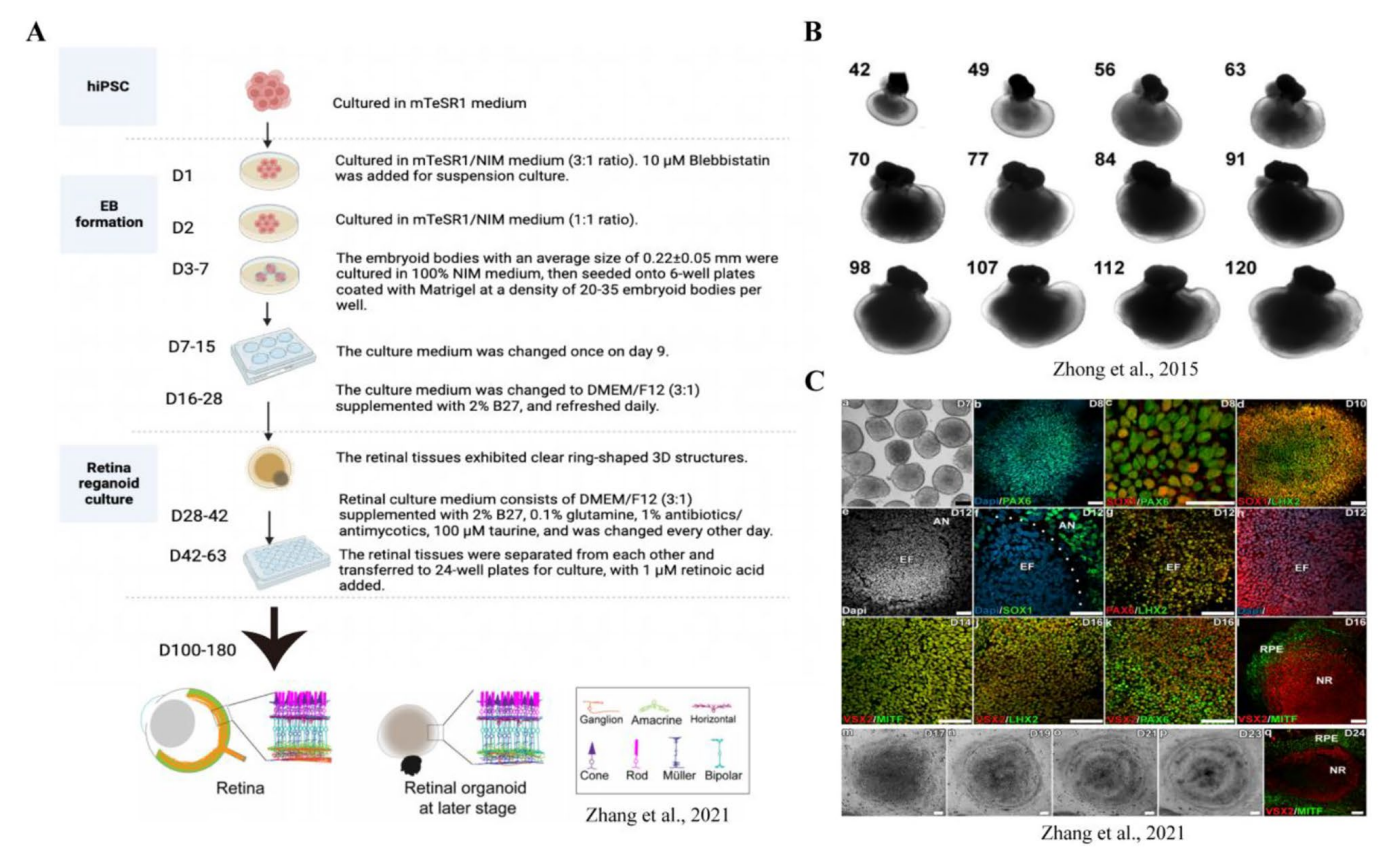
RO culture procedure. **A**, Culture procedure of RO, which after 180 days of culture closely resemble normal human developmental retina in terms of cell types and structural organization. **B**, Size, morphology, cellular organization, and steady growth of RO cultured for 42 to 120 days. **C**, hiPSC-derived retinal progenitors self-organized into eye field-like domains (EF) and subsequently differentiated into neural retina (NR) and retinal pigment epithelium (RPE). a-d, RO expressed different markers at different culture periods. Over time, neuroepithelial cells expressed SOX1 and PAX6 (b-c), retinal progenitor cells appeared in the center of aggregates expressing LHX2 (d). e-q, Later, cells in the eye field-like domains expressed VSX2 and MITF (e-h), and the NR domain gradually acquired an optic cup-like shape (h-q)

In addition, Lowe et al. used small hESC aggregates embedded in Matrigel to form single lumen epithelial cysts, which then adherently formed neuroepithelial colonies. Dispase treatment facilitated the detachment of these colonies to form laminated, mature RO in suspension culture [29]. All three methods have successfully employed both hESCs and hiPSCs and continue to undergo refinements and optimizations [24–26, 30–33]. hiPSC RO recapitulate human fetal retinal structure (Fig. 1).

## High-throughput Drug Screening for Retinal Organoids

The development of therapeutic drugs for retinal diseases begins with the establishment of high-throughput drug screening platforms. High-throughput screening is typically defined as screening over 10,000 compounds per day, while ultra-high-throughput screening refers to over 100,000 compounds per day. As a 3D culture model that closely mimics the complex physiology and pathology of the in vivo retina, RO can not only reflect individual variations among patients, but also allow large-scale directed differentiation and in vitro culture. Therefore, RO models have important application prospects in high-throughput drug screening for retinal diseases. However, mature high-throughput drug screening platforms based on RO have not yet been reported, mainly due to technical bottlenecks in tissue engineering and pharmacological evaluation, which makes it difficult to meet the demands of large-scale drug screening. These technical limitations include biomaterial selection for bioreactors to maintain an in vivo-like microenvironment, control of drug penetration and diffusion kinetics within tissues, quantitative detection of multicellular interactions, and parsing drug responses from different cell populations.

Specifically, as multicellular aggregates, RO have complex drug target sites, requiring consideration of drug bioavailability in the intricate microenvironment; different cell surface receptors may confer variable sensitivity to the same drug; intercellular signaling and feedback mechanisms can also affect the integrated drug response. In addition, in vitro culture of RO necessitates physiologic conditions akin to in vivo, such as temperature, pH, nutrient supply and waste removal, which are vital for maintaining normal tissue structure and function. Current high-throughput screening platforms still have many constraints in these aspects. With the advancement of microfluidic chips, bioprinting [34], and pharmacological evaluation techniques, the application prospects of RO models in high-throughput drug screening are highly promising.

Imaging analysis of RO 3D culture systems, unlike 2D cell screening platforms, is complicated by the presence of multiple cell types in specific and biologically relevant arrangements. Considerations such as spatial focusing/resolution, fluorophore/laser penetration imaging, drug diffusion kinetics, and parsing measured values from the various existing cell types make measuring experimental variables more challenging in these systems. To address this, researchers recently developed a fluorescence reporter-based screening platform [35] to evaluate mitochondrial health of RO photoreceptors in longitudinal studies, providing reliable quantitative and qualitative means for high-throughput drug screening.

Additionally, other technologies applied to 3D systems for high-throughput drug screening could be adapted to RO, thereby expanding output measurements and screening capabilities of these models. For example, luminescence analysis has been utilized to detect cell viability in high-throughput drug screening of tumor organoids derived from human induced pluripotent stem cells [36]. These technologies could seamlessly integrate with RO to develop screening platforms, although their reliance on lysing cells means measured outputs will inevitably represent tissue-wide rather than cell type-specific responses. Image-based high-throughput screening technologies are also being adapted to 3D systems, showing promise for RO-based screening platforms [37]. Finally, development of multi-electrode arrays now enables simultaneous electrophysiological recordings from hundreds of cells within tissues, and have been applied to RO systems [38].

The retina consists of highly complex layers of cells and extracellular matrix. RO RO derived from human embryonic stem cells (hESCs) or human induced pluripotent stem cells (hiPSCs) recapitulate some of the biological complexity of the retina. To simulate more complex physiological functions of retinal cells, such as angiogenesis and cell-cell interactions, retinal organoid-on-a-chip techniques have been developed in recent years. Retinal organoid-on-a-chip involves coculture of 7 types of hiPSC-derived retinal cells, enabling modeling of anterior segment diseases like dry eye syndrome as well as posterior segment conditions including age-related macular degeneration, diabetic macular edema, diabetic retinopathy, and glaucoma [39]. Culturing retinal organoids-on-a-chip in microfluidic systems is an effective approach for high-throughput screening, with one study confirming the applicability of ROCs for drug testing by replicating the retinal side effects of the antimalarial chloroquine and the antibiotic gentamicin [40]. Currently, by combining with modern 3D printing technology [34], RO can be mass-produced at high-throughput, providing powerful technical and platform support for exploring retinal pathogenesis and high-throughput drug screening [41].

## Drug Toxicity and Efficacy Evaluation for Retinal Organoids

Retinal drug toxicity detection is critical for developing safe therapies for many diseases. Human pluripotent stem cell (hPSC)-derived RO provide a suitable platform for preclinical drug toxicity assays, as they closely resemble the human retina and are easy to generate at scale [42]. Human PSC-derived ROs are a more bona fide model of human disease versus animal and 2D cell culture models [43, 44]. RO models can be leveraged to evaluate targeted therapies and drug toxicity during preclinical drug development [45]. RO were first applied to screen 133 FDA-approved drugs, selecting candidates based on cytotoxicity and potency, and comparing efficacy or toxicity of candidates versus clinically used drugs. This study showed RO retained genomic features of parent tumors, with Sunitinib exhibiting potent cytotoxicity towards both classic RB1-deficient and novel MYCN-amplified RBs, inhibiting RB proliferation while inducing differentiation [14]. Researchers have treated RO with well-known retinotoxic drugs including kainic acid, digoxin, tinidazole, sildenafil, ethanol and methanol, showing similar drug effects as reported in in vivo models and humans, providing strong evidence they are suitable for toxicology studies [42]. Additionally, mature human RO have been leveraged as a toxicity model to screen potential drug treatments for the age-related retinal degenerative disease MacTel2. This organoid toxicity model successfully identified an FDA-approved drug, fenofibrate, able to prevent photoreceptor death. This platform can easily be adapted to test any number of metabolic stressors and potential pharmacologic interventions for future therapeutic discovery in retinal disease [46]. This confirms RO toxicity assays are a directly implementable finding, by testing highly disease-relevant models. RO also serve as an important tool to study retinal development, with toxicity of the brominated flame retardant PBDE congener BDE-47 during early retinal development again confirmed using RO [47]. Intraocular drug delivery and providing targeted, sustained, controlled release using nanomedicine is one of the most challenging and popular topics in ocular drug development and toxicology assessments. With improvement and development of retinal organoid-on-a-chip techniques, these have been utilized to establish in vitro models mimicking intraocular delivery and develop next-generation retinal drug delivery strategies [11].

## Limitations of Retinal Organoids

Currently, heterogeneity is observed between different RO protocols as well as within and between individual organoids [13, 38]. This may be due to epigenetic memory from the starting somatic cells, which could promote or inhibit differentiation of induced pluripotent stem cells towards specific lineages [48]. Other challenges for RO include poor and variable maturation states of photoreceptors, and lack of direct contact with retinal pigment epithelium, leading to low responsiveness of organoids to light stimulation [49]. In addition, aging effects that underlie progressive neurodegeneration and late-onset retinal degenerative diseases may not manifest in current RO protocols even with prolonged culture [50]. However, continued improvements of protocols and establishment of new techniques, such as retinal-on-a-chip devices and co-culture systems [40], as well as the potential to induce aging through overexpression of progerin or telomere shortening, may enhance efficiency, reproducibility and maturity of organoids to alleviate some of these limitations [51, 52]. Additionally, unbiased omics studies, including proteomics, and rigorous measurements of neural activity are warranted to establish variability and functionality of organoids.

RO technology has great potential in modeling retinal development and disease, but currently still faces some limitations. First, RO can reconstitute multiple retinal cell types through directed differentiation in vitro, recapitulating the in vivo microenvironment, serving as an ideal model to study retinal development and disease mechanisms [53]. Compared to animal models, organoids can reflect human retinal development more faithfully, yielding research findings with greater clinical translational value [25]. This is significant for unraveling principles of retinal development and disease pathogenesis. However, currently heterogeneity is observed between different RO protocols as well as within and between individual organoids [13, 38]. This may be due to epigenetic memory from the starting somatic cells, which could promote or inhibit differentiation of induced pluripotent stem cells towards specific lineages [48]. Current RO also have some defects in cell maturation and tissue structural integrity. For example, photoreceptor differentiation and maturation are incomplete, with limited expression of photopigments, and weaker light responsiveness [49, 54]. Organoids lack a monolayer structure with close apposition of photoreceptors and retinal pigment epithelium like the in vivo retina [55, 56]. These limitations impede applications in modeling physiological processes like outer segment formation and the visual cycle.

Moreover, current RO have difficulty recapitulating the progressive aging changes of the human retina during development and disease [50, 57], which are especially important for modeling late-onset retinal degenerative diseases. Other limitations pertain to normal development of certain cell types in organoids, namely retinal ganglion cells, immune-related cells, and the vascular system [57]. Although retinal ganglion cells can develop in organoids, they gradually diminish with organoid maturation as survival of ganglion cells depends on connecting with cortical neurons. Incorporating these cell types will require assembloid techniques to combine cells from different developmental lineages. At least some of these limitations are being addressed through use of organoid-retinal pigment epithelium co-cultures and retinal-on-a-chip technologies [40]. Methodologically, currently most rely on morphological analysis to assess differentiation in RO, lacking direct assay of physiological function and neural activity, limiting application prospects as pharmacological or toxicological screening platforms. Cell sources and culture protocols used by different laboratories have some heterogeneity, with reproducibility of outcomes that needs improvement [58].

Establishing standardized culture protocols, incorporating aged cell models, enriching functional assays will all help enhance maturity and utility of RO [25]. Continued improvements of protocols and establishment of new techniques, such as retinal-on-a-chip devices and co-culture systems [40], as well as the potential to induce aging through overexpression of progerin or telomere shortening, may enhance efficiency, reproducibility and maturity of organoids to alleviate some of these limitations [51, 52]. Additionally, unbiased omics studies, including proteomics, and rigorous measurements of neural activity are warranted to establish variability and functionality of organoids. With further maturation of stem cell differentiation and tissue engineering technologies, RO hold promise to become a powerful tool for drug discovery and disease modeling to shed new light on treatment of retinal disorders. But for now, there are still aspects of the technologies that need continued optimization and refinement before organoid applications can move deeper into facilitating clinical translation.

## Next-generation Retinal Organoids

**Fig. 2:**
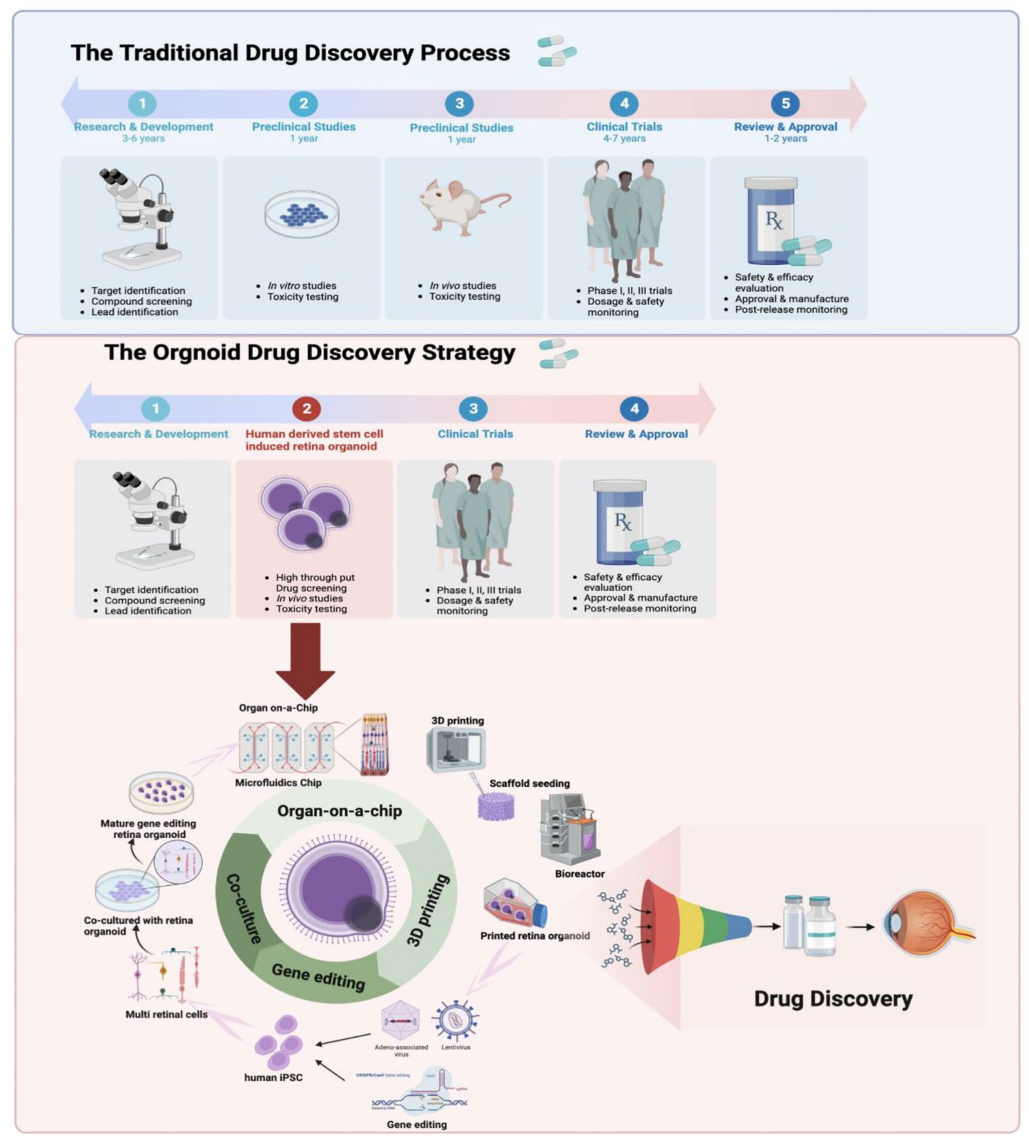
The drug discovery strategy using the next generation of retina organoid

The development of RO technology brings new hope for the treatment of retinal diseases. In recent years, RO culture systems derived from human induced pluripotent stem cells have demonstrated tremendous potential in disease modeling and drug screening. However, there are still limitations to this technology, including incomplete cellular composition and immature functional responses. Therefore, designing optimized culture strategies to facilitate RO towards higher levels of maturity is an urgent problem to be solved in this field. This article will explore the next generation of RO culture strategies and research directions from two aspects: optimizing culture methods and utilizing organ-on-a-chip techniques(Figure 2).

### Optimizing Retinal Organoid Culture Methods

Current RO cultures mainly utilize the suspension aggregate differentiation method of induced pluripotent stem cells, which suffers from issues like hindered nutrient diffusion, hypoxia, and cell death, leading to incomplete differentiation and simple cellular composition. Therefore, optimizing culture methods is an important strategy to enhance RO maturity.

#### Incorporating More Retinal Cell Types

Existing RO primarily contain photoreceptor and ganglion cells, but lack vasculature and immune cells, which is a major limitation [25]. Therefore, adding cell types like vascular endothelial cells and macrophages would aid in reconstituting the cellular composition and immune microenvironment of the retina [59, 60]. Retinal pigment epithelial cells can also be incorporated to construct a complete retinal system [24]. Moreover, co-culturing different cell types can also promote maturation of retinal cells. For instance, co-culture of retinal ganglion cells with photoreceptor cells can improve the viability and functionality of photoreceptors [49]; co-culture with vascular endothelial cells enables observing the effect of angiogenesis on retinal cell differentiation [53, 59, 60]. Some studies utilize brain microvascular endothelial cells and neural progenitor cells in co-culture, and find induced expression of pro-angiogenic VEGF can promote neuronal differentiation. To better mimic human BBB, a 3D microfluidic BBB model was designed, consisting of microvascular networks self-assembled from human iPSC-derived endothelial cells, primary cortical neurons and astrocytes, which dynamically reproduces the BBB structure and direct cellular interactions [61]. Similarly, in stem cell-derived RO, co-culturing strategies of multiple cell types can better assemble the multilayered architecture of the retina and promote its differentiation and maturation. Additionally, immune cells like microglia have also been shown to help maintain the stability of RO [62].

#### Optimizing Differentiation Induction Protocols

Current organoid cultures mostly use empirical differentiation protocols, resulting in large variability between different labs. Further systematic optimization of conditions like concentrations and timing of differentiation factors will aid in reducing batch-to-batch and cell line variability, improving reproducibility of differentiation. To systematically refine and optimize RO differentiation protocols, integrated design from multiple aspects is required. Firstly, quantitative analysis of expression patterns of various induction factors like FGF, IGF etc., during retinal development should be performed to determine their temporal windows and dose-response relationships. This can be achieved by dynamic monitoring of factor expression levels [63]. Next, small molecule compounds specifically targeting multiple key signaling pathways governing retinal development can be screened to accurately activate or inhibit pathway activity [64]. Additionally, gene editing tools like CRISPR/Cas9 can be utilized to knock out or overexpress transcription factors controlling retinal development, directly manipulating gene expression to guide differentiation [25]. In designing induction regimens, staged induction strategies can be adopted based on the above quantitative analysis data: initial maintenance of pluripotency with FGF etc., [65] followed by transition to pro-differentiation factors like IGF, TGFβ etc., [24] along with precise modulation of signaling pathways with specific small molecules [66], and overexpression of key transcription factors driving differentiation [63]. Throughout the induction process, molecular markers of retinal development like Pax6, Chx10 etc., [64] should be closely monitored to provide feedback for adjusting regimens. Moreover, stem cells from different sources may exhibit differential responses to differentiation cues, necessitating optimization specific to each cell type [67]. Additionally, high-throughput microfluidic differentiation platforms with automated operation can be established to extensively test the effects of various conditions to rapidly define optimal protocols [16]. In summary, systematic design and optimization of RO differentiation induction requires integration of multi-disciplinary knowledge in bioinformatics, stem cell engineering, molecular biology etc., to enable standardization and maturation of this technology. This will greatly improve the reliability and reproducibility of RO generation.

#### Prolonging Culture Time

Current RO cultures are often around 100 days, leading to immature tissue development and imperfect cell functionality. To obtain higher-order retinal tissues, prolonging culture time is an effective approach. Relevant studies have shown that extending culture time can significantly enhance RO maturity. Extending the culture time is key to obtaining mature, multi-cellular RO. Current studies have adopted various strategies to prolong the culture time of RO. The culture duration directly affects the maturity of RO structure and function. Studies show that short-term culture of 35–60 days allows observation of early retinal development, including formation of some photoreceptor cells, but outer segments remain immature, cellular connections are incomplete, and light responses are weak [25, 68]. In comparison, extending the culture to 130–200 days yields more mature RO. These can form more intact layered architecture of the retina, elongated photoreceptor outer segments, increased visual pigment accumulation, and higher degrees of maturity in non-neural cells, all of which support and enhance light responses [38, 53]. In summary, prolonging the culture time is critical for generating mature RO, which is important for establishing reliable models of retinal development and disease.

In summary, prolonging RO culture time is essential for achieving higher degrees of cell maturity and more refined tissue architecture. Most current studies extend culture to 140–200 days, but theoretically even longer periods could bring them closer to fully mature retina. Future efforts may explore even longer cultures, or dynamic condition-switching strategies, to better recapitulate the full developmental timeline, which will aid in meeting the high standards required for clinical therapy.

#### Incorporating Tissue Engineering Techniques

Traditional RO cultures use suspension droplet culture, limiting tissue size and structure and causing issues like malnutrition and hypoxia. Incorporating tissue engineering techniques can significantly enhance RO tissue morphology and function. 3D bioprinting can also precisely control cell positioning to reconstruct retinal layered structure [34].

One approach is engineering 3D scaffolds for RO. Assawachananont et al. designed layered porous scaffolds to guide growth of retinal cells in developmental order, forming stratified retinal tissues. Sun et al. developed a novel 3D printed polydimethylsiloxane (PDMS) microwell platform that enables one-step formation and long-term culture of RO with maintained homogeneity and promoted maturation without the need for BMP4 and Matrigel, establishing a xeno-free workflow amenable for retinal disease modeling, drug screening and clinical translation [34].

There is a range of three-dimensional tissue engineering and biofabrication technologies that allow precise control over the final material’s location, incorporating many different cell types and surrounding cell-derived extracellular matrix (ECM) to mimic living tissues. The focus in the development of these technologies has been on technical capabilities. One approach is engineering 3D scaffolds for RO. Assawachananont et al. designed layered porous scaffolds to guide growth of retinal cells in developmental order, forming stratified retinal tissues. Sun et al. developed a novel 3D printed polydimethylsiloxane (PDMS) microwell platform that enables one-step formation and long-term culture of RO with maintained homogeneity and promoted maturation without the need for BMP4 and Matrigel, establishing a xeno-free workflow amenable for retinal disease modeling, drug screening and clinical translation [34]. Using biomaterial scaffolds or microfluidic chips for dynamic perfusion culture can improve nutrient supply and increase cell viability. The latest advances in 3D culture technologies and tissue engineering enable 3D organoids to partially recapitulate the anatomical architecture, biological complexity, and physiology of different tissues [69], while addressing the obvious problem of limited cell availability encountered in other approaches [70, 71]. In particular, in vitro stratified RO have proven to mimic the spatiotemporal development of native retinal tissue in a manner not observable in animal models [72]. RO technology has revolutionized the field of ophthalmic science by not only providing advanced in vitro study models but also enabling the generation of clinically relevant numbers of retinal cells for transplantation therapy for the first time [73].

Retinal cell bioprinting technology still has limitations. Importantly Masaeli et al. Utilizing inkjet bioprinting technology, a functional rpe-photoreceptor system was generated using a scaffold-free approach [74–76]. While the results are promising, tissue engineering techniques have yet to demonstrate a variety of different printing capabilities. It is foreseeable that some of the current limitations of three-dimensional tissue engineering systems can be addressed through so-called “microfluidic” or “lab-on-a-chip” platforms. Such systems allow single cell micro-scale positioning in a highly complex two-dimensional system, breaking the inherent inaccessibility of complexity common to most 3D tissue engineering strategies. Microfluidics has the potential to quantitate biological processes at the single cell level and high temporal resolution [77]. Microfluidic technologies have been applied for RO culture. Marcos et al. developed microfluidic differentiation chips enabling continuous dynamic differentiation and visual tracking of retinal development [16]. These integrated systems control environments and nutrient delivery for automated culture.

Overall, innovating RO culture methods using tissue engineering for scaffolds, co-culture systems, and microfluidics can tremendously improve tissue structure and function, expediting technological maturation and application. Further research warrants deeper investigation in this area.

### Applying Organ-on-a-chip Technology for Retinal Organoid Culture

Retinal organ-on-a-chip is an emerging in vitro model that highly mimics the structure and function of human retina by integrating RO and chip technology. This model overcomes the limitations of animal models and can accurately reproduce the process of drug delivery in the human eye for drug efficacy evaluation and disease modeling [41]. Compared with traditional static cultivation, the chip system enables precise control of environmental factors, which is beneficial to standardized production of high-quality RO. Current research is adopting strategies such as multi-layered tissue co-culture and vascular network construction to reconstruct the physiological structure of the retina on an integrated chip platform [40]. In addition, RO derived from induced pluripotent stem cells provide a reliable cell source for constructing disease models and drug screening platforms [11]. Future research will continue to optimize nutrient perfusion systems and sensor detection functions in order to achieve more precise simulation of the in vivo microenvironment, promoting basic research and clinical translation of retinal diseases [34]. In summary, retinal organ-on-a-chip technology integrates multidisciplinary innovations and demonstrates great application potential.

#### Microfluidic Nutrient Supply for Organ-on-a-chip

Microfluidic nutrient supply is a very important part of organ-on-a-chip technology. Recent studies have adopted various innovative strategies to achieve microfluidic nutrient supply for organ chips. For example, the PDMS microwell chip platform fabricated by 3D printing technology can enable uniform and stable nutrient supply, which is beneficial for long-term maintenance of RO. In addition, micro-millifluidic bioreactors can provide shear-stress-free steady-state flow to maintain the physiological structure and function of RO [78]. Moreover, constructing vascular networks makes perfusion of nutrients possible, which is the key to mimicking the in vivo environment [40]. Future research may continue to optimize microfluidic systems, such as introducing sensors to monitor nutrient consumption and dynamically adjusting perfusion parameters based on feedback, thereby achieving precise control over nutrient supply [11]. In summary, microfluidic nutrient supply technology provides important support for the long-term stability of organ chips and is an indispensable part of mimicking the in vivo microenvironment.

#### Multi-cell Co-culture for Organ-on-a-chip

The retina is composed of multiple complex cell types, and stem cell-derived RO cannot fully induce all cell types. Therefore, incorporating different retinal cell types into organoid culture systems is a relatively mature approach. To better mimic the human blood-brain barrier, a 3D microfluidic BBB model was designed, consisting of microvascular networks self-assembled from human iPSC-derived endothelial cells, primary cortical neurons and astrocytes, which dynamically reproduces the BBB structure and direct cellular interactions [79]. Microglia are tissue-specific resident macrophages and play a critical role in retinal development and homeostasis [80, 81]. Recently, Gao et al. derived microglia from human embryonic stem cells which can promote proper localization and function of microglia when co-cultured with human RO, providing new tools for “integral retinal organs” to facilitate research on retinal development, diseases and therapeutic screening [82].

A significant advantage of microfluidic chips is the ability to integrate multiple cell culture chambers for co-culturing different cell types to reconstitute the multicellular retinal microenvironment. For example, Shi et al. generated vascularized human cortical organoids (vOrganoids) by co-culturing human embryonic stem cells or human induced pluripotent stem cells with human umbilical vein endothelial cells in vitro [83]. Results showed that neurons in vOrganoids were more mature, with enhanced neurite or axon growth, and expressed fewer apoptotic or hypoxic markers, while non-vascularized organoids did not. Although the retinal vascular system is completely different from the brain vascular system, applying similar approaches to RO culture may alleviate oxygen supply and nutritional support deficiencies to some extent, given the limitations of conventional organic culture. This holds potential for improving the maturity and functional development of retinal cells, especially photoreceptors, in RO culture. A groundbreaking study reported combining the self-assembling capabilities of ROs with the precision controllable assembly and measurement of microfluidic platforms. This work proposed a novel device as a microphysiological model of the human retina, where limitations of lack of vascularization and co-localization with RPE were successfully addressed [40]. Yeste et al. developed a novel microfluidic device whereby cells are arranged in parallel compartments but are highly interconnected through a grid of microgrooves located under the cells. They co-cultured primary human retinal endothelial cells, a human neuroblastoma cell line and a human RPE cell line to model the tissue-tissue interface of the retinal blood-barrier [84].

#### High-throughput Drug Screening for Organ-on-a-chip

Dynamic drug screening is an important application of organ-on-a-chip technology. Recent studies have adopted various strategies to achieve dynamic drug efficacy assessment on organ chip platforms [11, 40, 77, 78, 85]. For example, RO chips derived from induced pluripotent stem cells can serve as a new translational model to validate gene therapy vectors for the retina [85]. In addition, microfluidic reactors enable continuous perfusion of drugs, simulating in vivo pharmacokinetics for drug screening [78]. Moreover, multi-layered tissue construction lays the foundation for evaluating drug transport between different cells [40, 78]. Future research may continue to optimize the sensing and detection functions of organ chips and develop high-throughput drug screening platforms, achieving real-time monitoring of pharmacodynamic processes [11]. In summary, dynamic drug efficacy assessment is an important application of organ-on-a-chip technology, providing more human-relevant screening models for drug discovery.

In summary, RO technology still has some way to go for full maturation. Future work can improve culture quality by optimizing differentiation protocols, extending culture periods, and incorporating tissue engineering techniques. Additionally, constructing dynamic retinal-on-a-chip models leveraging microfluidic and integrated sensors from organ chips will aid in advancing the technology towards greater maturity and utility. This provides better platforms for precise disease modeling, drug screening, and discovering potential therapeutics. With continued research, RO technology is sure to shed new light on preventing and treating retinal diseases.

## Conclusion

In summary, RO technology has rapidly advanced in recent years to become a valuable tool for modeling retinal development and disease, facilitating drug screening, and evaluating drug toxicity and efficacy. Protocols for generating RO from PSCs have improved to yield complex, laminated structures containing most retinal neuron types [25, 86]. Microfluidics, bioengineering approaches, and co-culture systems have enhanced organoid maturation and architecture to better recapitulate native retinal tissues [38, 87]. High-throughput screening platforms utilizing RO have enabled rapid testing of drug libraries to discover promising therapeutics [88]. Microfluidic organ-on-a-chip models have allowed sophisticated drug testing under controlled microenvironments [89]. Several studies have demonstrated human RO can effectively model multiple retinal diseases and predict efficacies of drugs that showed translational potential in clinical trials [90–93].

However, limitations remain with current RO systems. While protocols generate key retinal cell types, the completeness of retinal differentiation and organization falls short of native retina. Immature organoids lack neuronal maturation and photoreceptor outer segment formation critical for visual function. Culture periods are restricted, limiting long-term modeling applications. Variability between organoid batches hampers screening reproducibility. Next-generation organoid engineering approaches seek to address these limitations through innovative biomaterials, bioreactors, co-culture, and microfluidic systems to generate enhanced organoids [15–20]. Quadruple-layered retina-on-a-chip devices with RPE have achieved improved morphology [40], while bioprinting holds promise to pattern cells and matrix into higher order structures [94].

Advancing organoid maturity and consistency will expand utility for disease modeling, drug discovery, and personalized medicine applications. Coupling advanced tissue engineering techniques like microfluidics and bioprinting with stem cell biology and gene editing will enable unprecedented control over organoid development, architecture, and function. Leveraging organoid banks from diverse genetic backgrounds will facilitate screening in defined genetic contexts. Stem cell-derived organoids have tremendous potential to transform retinal disease research and therapy, but continued progress in organoid culture systems is key to fully realizing this potential.

## Data Availability

This article will be accessed on online upon publication.
